# Development and diapause induction of the Indian meal moth, *Plodia interpunctella* (Hübner) (Lepidoptera: Pyralidae) at different photoperiods

**DOI:** 10.1038/s41598-020-71659-7

**Published:** 2020-09-07

**Authors:** Md. Mahbub Hasan, Sayla Aktar Chowdhory, A. S. M. Shafiqur Rahman, Christos G. Athanassiou

**Affiliations:** 1 Department of Zoology, Rajshahi University, Rajshahi, 6205 Bangladesh; 2 Laboratory of Entomology and Agricultural Zoology, Department of Agriculture, Crop Production and Rural Environment, University of Thessaly, Phytokou Str., 38446 Nea Ionia, Magnesia Greece

**Keywords:** Entomology, Animal physiology, Biochemistry, Zoology

## Abstract

Diapause concerns the fascinating phenomenon in the biology of insect development which allows better understanding the local adaptation and phenotypic plasticity to seasonal variations in environment. There is lot of reasons to carry out the research on diapause both for fundamental and applied sciences. Photoperiod is one of the main environmental cues followed by insects to predict the forthcoming seasonal changes and to adapt these changes in their life-history traits. Thus, the effect of different photoperiod regimes on development and diapause induction of larvae of the Indian meal moth *Plodia interpunctella* (Hübner) was evaluated at a constant temperature of 17 °C. Development was significantly faster at a photoperiod of 12:12 light:darkness (L:D) than at 8:16, 10:14, 14:10 and 16:8 L:D. A photoperiod of 12:12 (L:D) induced most larvae (≥ 71%) to enter diapause, while this percentage was slightly lower (60%) at both shorter(8 h) and longer (16 h) day lengths (50%). The different photoperiod regimes did not affect the percentage of adult emergence. Fat and protein composition of the diapausing larvae differed significantly among treatments as well as between diapausing and non-diapausing larvae. Larvae developing from 8:16 (L:D) contained the maximum amount of protein (36.8%) compared to other regimes, while the minimum amount (21.0%) was noted in larvae that developed at 16:8 (L:D). Six types of fatty acids were detected in the larvae: myristic acid (methyl tetradecenoate), palmitoleic acid (9-hexadecenoic acid, methyl ester), palmitic acid (hexadecenoic acid, methyl ester), linoleic acid (9, 12-Octadecadienoic acid (Z, Z), methyl ester), oleic acid [9-octadecenoic acid, methyl ester (E)] and stearic acid (octadecanoic acid, methyl ester). The results also reveal that the percent of fatty acids detected in the diapausing larvae varies significantly and the same trends imply in the interaction of fatty acid and photoperiod regimes. Moreover, three quarters of the total variance was accounted for by the Principal Component Analysis (PCA) of the fatty acids. Different proportions of fatty acids were noted among treatments, suggesting that photoperiod influences a number of key biological traits in *P. interpunctella*, much more than the percentage of the diapausing larvae per se.

## Introduction

Insect development or reproduction is being frequently halted by inadequate resources or harsh environmental conditions. Dormancy plays a prime role to minimise the stresses of these unfavourable intervals and stabilise the insect life cycles with favourable periods^1^. Dormancy response may include both pre-programmed, endocrine-mediated diapause, and also quiescence induced directly by the adverse environmental conditions including the low temperature, drought, insect density etc. Quiescence represents a halt in insect development by unfavorable conditions that can occur in any life stage. In contrast with quiescence, diapause exhibits only during a specific developmental stage that may be either facultative (occurring in response to environmental cues) or obligate (occurring in each generation regardless of environmental cues). Developmental time is one of the life-history traits known to be at least partly under photoperiodic control in several insect species^1,2^, and many insects enter diapause in response to photoperiod^1,3^. Photoperiod is the most reliable indicator for timekeeping in organisms^4–6^, and many studies have probed the mechanisms of biochronology^7–9^. Most insects are able to regulate their biological cycles utilizing diapause, which is a characteristic that can be taken into account in designing detection and control strategies. In the case of many stored product insect species, which, theoretically, develop in an enclosed environment that are not characterized by a regular succession of photophase and scotophase, diapause induction has substantial differences in comparison with field pests^10^.

The Indian meal moth, *Plodia interpunctella* (Hübner) (Lepidoptera: Pyralidae) is an important stored product moth, with an extremely large variety of food preferences, causing serious losses and degradations^11,12^. Several surveillances indicate that *P. interpunctella* has a global distribution, and it is probably the most common stored product moth species at the post-harvest stages of grains and related amylaceous products^11^. *Plodia interpunctella* larvae enter into a facultative pre-pupal diapause induced by photoperiod, temperature, strain of origin, or diet^10,13–15^. Diapause seems to be an important life stage for most stored-product Lepidoptera^13,14^. In *P. interpunctella,* the developmental arrest is more transient at temperatures within the developmental range than it is in typical temperate species^16^. Bell^17^ reported that *P. interpunctella* enters diapause at temperatures between 20 and 25 °C when the photophase is ≤ 13 h. Moreover, the behavioral changes in *P. interpunctella* late instar (mature) larvae may be triggered by decreasing day length. It has also been found that low temperatures are able to cause rapid diapause induction in *P. interpunctella* larvae^18,19^. Given that in most storage facilities temperatures are not controlled but instead follow the fluctuations of outside temperatures^20^, *P. interpunctella* usually enters diapause during the colder months^21^. At 20 °C diapause is quickly terminated at very long photophases, such as 16:8 (L:D)^15^. Occurrence of diapause in *P. interpunctella* has several implications regarding management of this species, given that diapausing larvae are much more tolerant to many of the commonly used insecticides in stored product protection. For instance, Bell^22^ found that diapausing larvae were 2–8 times more tolerant than non-diapausing larvae to phosphine. The same holds for non-chemical control methods that are used at the post-harvest stages of agricultural commodities, such as low temperatures^22^. This is particularly important, as diapausing larvae may not be controlled by methods that are usually effective for the control of non-diapausing *P. interpunctella* individuals, allowing a rapid population development after the termination of the treatment. At the same time, surviving diapausing larvae may remain undetected, due to their negligible mobility, providing the false impression that the application was effective.

Generally, the photoperiodic response of *P. interpunctella* is of a long-day type^23,24^. However, a number of papers have discussed the possibility of quantitative time measurement, i.e., a ‘‘clock’’ mechanism that distinguishes between actual day- or night- lengths, rather than merely discriminating ‘‘long’’ from ‘‘short’’, an idea that changes the basic theoretical approach for diapause induction (Kimura^25^ for *Drosophila testacea* Roser, Zaslavski and Fomenko^26^ for *Megoura viciae* Buckton, Numata^27^ for *Riptortus clavatus* (Thunberg), Hori^28^ for *Palomena angulosa* (Motschulsky), Kimura^29^ for *Drosophila auraria* Peng, Hardie^30^ for *Megoura viciae* Buckton, Spieth and Sauer^31^ for *Pieris brassicae* (L.). Moreover, the photoperiodism plays a primary role in regulating the induction of diapause since it is used almost universally by insects in different geographical areas, but there are serious interactions in its expression with additional parameters, such as temperature and rainfall^32^. There are several key components that are involved in the entire process of diapause induction through photoperiodism, such as the presence of photoreceptors capable for differentiating the photophase and scotophase, capacity for measuring the length of each photophase (or scotophase) and the ability to accumulate the number of short (or long) days^32^. The experimental methods which are being used to determine the qualitative or quantitative principle for measuring the photoperiodic time in insects at different short photophases include: (i) the incidence of diapause at various temperatures; (ii) the number of days required to induce 50% diapause and (iii) the intensity (or duration) of diapause. The measurement of photoperiodic time usually considered as qualitative when all these approaches showed insignificant differences while significant differences are exhibited as quantitative^33^. However, there is still inadequate data towards the above methods for *P. interpunctella*, especially in the case of low temperatures, which may cause a more rapid diapause induction.

Previous research has investigated the roles of environment^1,34^, molecular regulation^3,35,36^, circadian mechanisms^8,37,38^, photoreceptors and clock genes^39,40^, cold tolerance^41^, and climate change^42^ on diapause, while additional studies have investigated energy management in diapausing insects^43,44^. Turunen and Chippendale^45^ reported that substantial amounts of a diapause-associated protein (DAP) accumulate in the fat body of early diapausing larvae of southern cornstalk borer moth, *Diatraea crambidoides* (Grote). Tan et al*.*^46^ compared quantitatively the expression profiles of proteins in diapause preparation phase in the adult female cabbage beetles, *Colaphellus bowringi* Baly and a total 3,175 proteins was identified. They also differentiated the composition of DAP from other types of fat composition while working on the proteomics in diapausing *C. bowringi*. It has been also reported that the amount of DAP usually varies within a species depending on the geographical region of origin. For instance, Kikukawa et al*.*^47^ observed that the fat body of *D. grandiosella* diapausing larvae originating in south-central Mexico (19°N latitude) contained substantially higher amounts of DAP than larvae from Missouri (37°N latitude). They also added that this DAP is synthesized and accumulated in the fat body at the beginning of diapause and depleted at the end of diapause. After that, it is released from the fat body and does not accumulate in the haemolymph of the diapausing larvae. The amino acid composition of DAP was found to have little resemblance to that of the free amino acids in the haemolymph^46^.

There are several functions of DAP suggesting it is a storage protein, an enzyme or proenzyme, a carrier or pro-carrier and a protectant^44,48–50^. It has been reported that DAP accumulates in the fat body after the completion of larval feeding, and it differs from the known hexameric storage proteins of feeding larvae of Diptera and Lepidoptera^51,52^. The pattern of DAP synthesis is similar to that of specific proteins synthesized in response to a stress stimulus such as a heat shock^53,54^. DAP protects larvae from harsh conditions that they are exposed to during their diapause, similar to the presumed role of heat-shock or stress proteins. In contrast, DAP is produced in advance of the environmental stress, unlike heat shock proteins which are synthesized in response to a stress stimulus^53^. *Plodia interpunctella* refers as one of the most cold hardy stored product pests^55^ and the diapausing larvae may spend six or more months in diapause^18,22^. The low temperature enhanced the response in diapause at short-day conditions. For example, the maximum (80%) diapause response was observed in flesh flies when reared at 25 °C while the diapause incidence is elevated to nearly 100% at 18 °C. In the present study, diapause induction of *P. interpunctella* was systematically investigated under laboratory conditions, at a constant low temperature level (17 °C), for which there are no data available, despite the fact that exposure of *P. interpunctella* to low temperatures can be further implemented for mass rearing of parasitoids^56^. This was carried out at different photoperiod regimes. In addition to diapause induction, we examined larval development, as well as the abundance of proteins and fatty acids in the larvae.

## Results

### Photoperiodic induction of diapause

**Figure 1:**
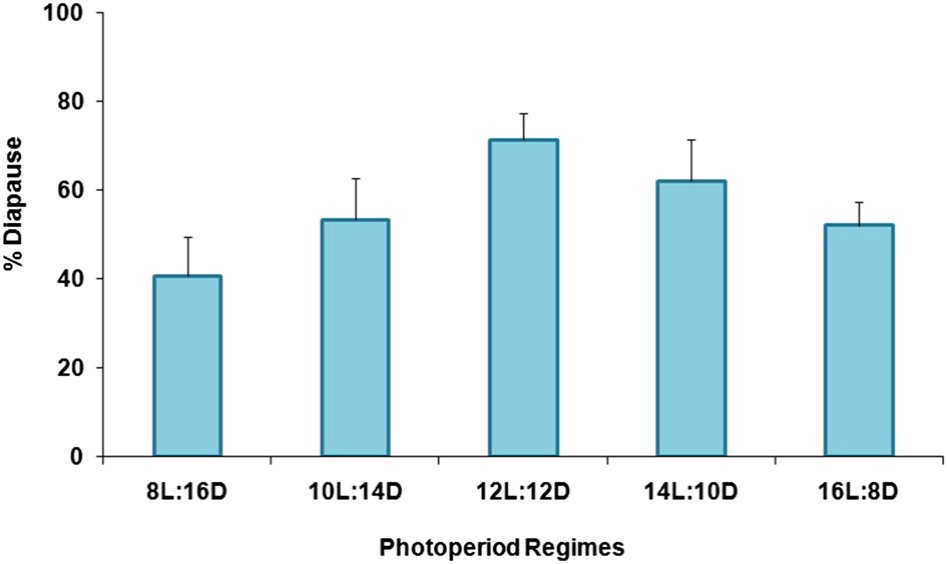
Percentage (% ± SE) of diapausing larvae of *P. interpunctella* at different photoperiods, at 17 °C.

**Figure 2:**
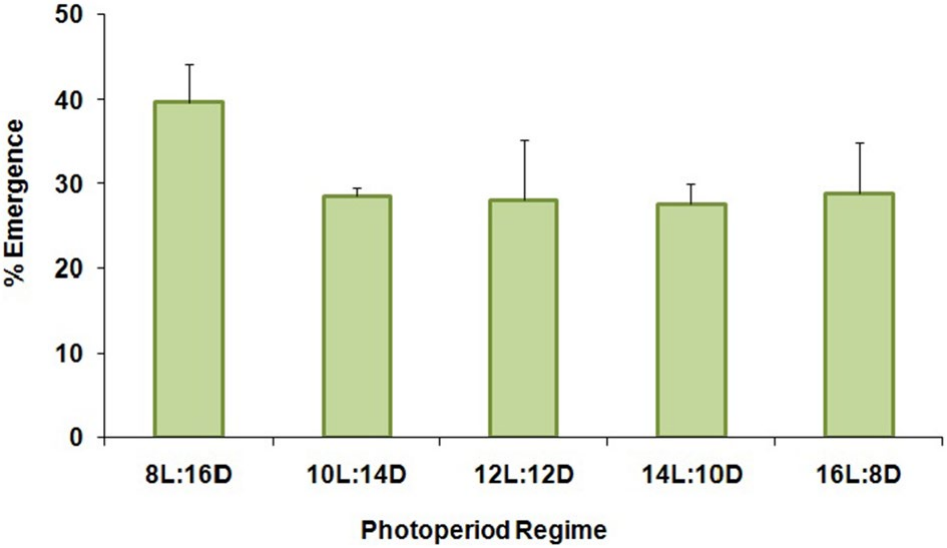
Percentage (% ± SE) of adult emergence in *P. interpunctella* larvae reared at 17 °C and different photoperiod regimes.

**Figure 3:**
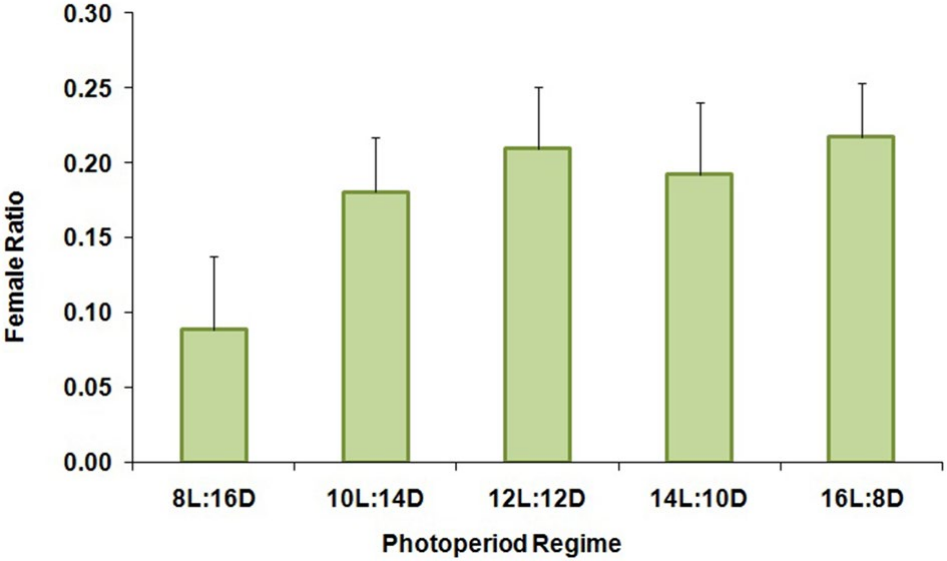
Female ratio (female:male ± SE) in adults that had been emerged from *P. interpunctella* larvae reared at 17 °C and different photoperiod regimes.

**Figure 4:**
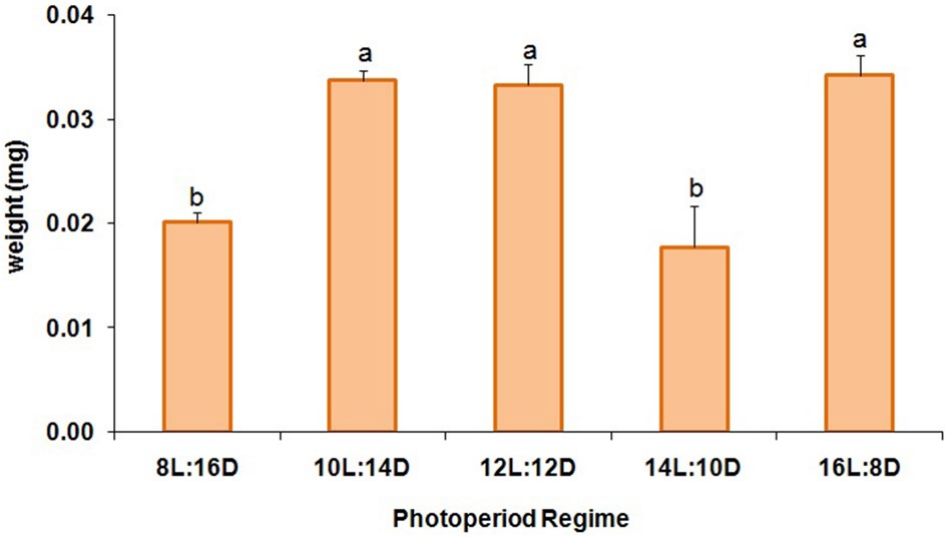
Larval weight (mg ± SE) of *P. interpunctella* larvae reared at 17 °C and different photoperiod regimes (means followed by the same letter are not significantly different; HSD test at 5%).

**Figure 5:**
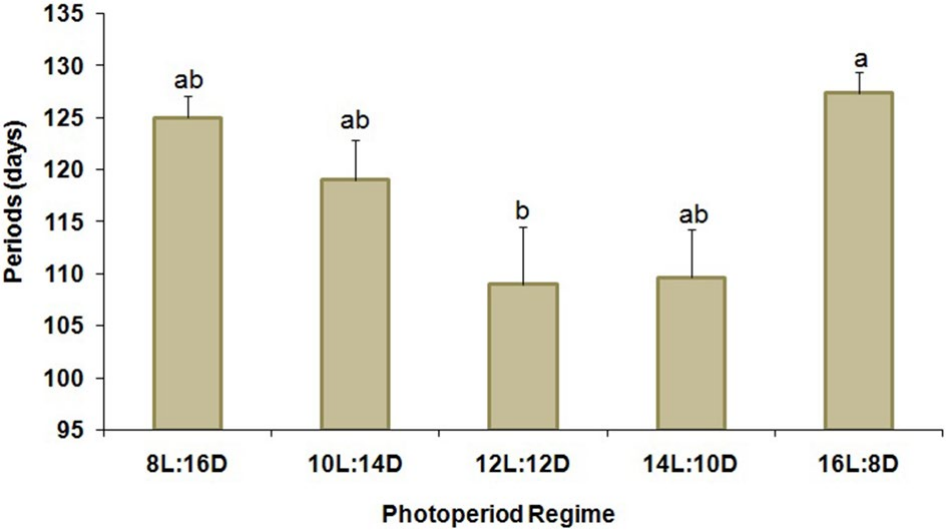
Developmental period (days ± SE) of *P. interpunctella* larvae that had been reared at 17 °C and different photoperiod regimes (means followed by the same letter are not significantly different; HSD test at 5%).

The percentage of larval diapause induction was not significantly affected by the different photoperiod regimes (F = 2.10; df = 4,10; P = 0.16) (Fig. 1). Numerically, the 12:12 (L:D) regime resulted in the highest percentage (≥ 71%) of diapausing larvae, while both longer and shorter photoperiods produced somewhat lower levels of diapause (Fig. 1). Nevertheless, there was no clear critical photoperiod shown in this experiment since the diapause incidence varied little among the photoperiods tested. Maximum adult emergence without diapause (39.70%) was noted for larvae that had been exposed to 8:16 (L:D) (Fig. 2) but there were no significant differences among treatments (F = 1.16; df = 4,10; P = 0.39). The ratio of females was also not significantly affected (F = 1.49; df = 4,10; P = 0.28) by the different regimes, and in all cases remained far below 1:1 (females:males) (Fig. 3). The lowest female ratio was recorded at 8:16 (L:D), where it was < 0.1. Larval weight differed significantly among treatments (F = 3.19; df = 4,10; P = 0.03) (Fig. 4), with lowest weights noted for larvae that had been exposed to 8:16 and 14:10 (L:D). Larval developmental period varied significantly among the different photoperiod regimes (F = 4.82; df = 4,10; P = 0.02), with a maximum larval developmental period of 127.33 d at 16:8 (L:D) (Fig. 5).

### Biochemical profiles

#### Protein profiles

**Figure 6:**
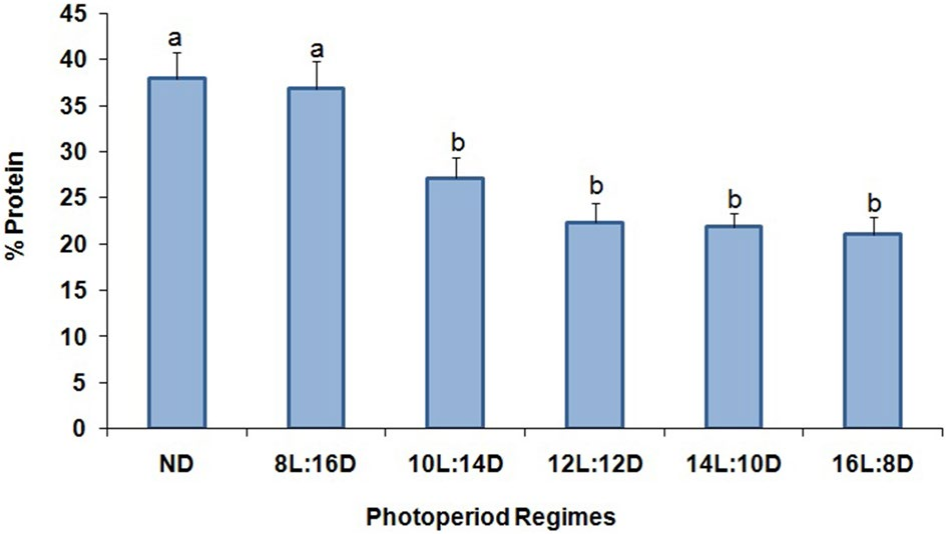
Percentage (% ± SE) of total protein in *P. interpunctella* larvae reared at 17 °C and different photoperiod regimes (means followed by the same letter are not significantly different; HSD test at 5%).

Significant differences were noted in the percentage of protein in larvae among the different photoperiod regimes (F = 44.6; df = 5,12; P < 0.001), ranging from 36.8 to 21.0% for 8:16 to 16:8 (L:D) (Fig. 6). Larvae reared at 8:16 (L:D) had a similar percentage of protein as non-diapausing larvae. Moreover, the increase of the photophase to 12, 14 and 16 h also showed similar trends of protein percentage (Fig. 6).

#### Fatty acid profiles

Results clearly revealed that there was a significant (F = 29.61; df = 11,30; P < 0.001) impact of photoperiodic response on the fatty acid profiles in diapausing *P. interpunctella* larvae (Table 1). We detected six types of fatty acids, i.e. myristic acid (methyl tetradecenoate), palmitoleic acid (9-hexadecenoic acid, methyl ester), palmitic acid (hexadecenoic acid, methyl ester), linoleic acid (9, 12-octadecadienoic acid (Z, Z), methyl ester), oleic acid [9-octadecenoic acid, methyl ester (E)], and stearic acid (octadecanoic acid, methyl ester) (Table 1). Methyl stearate and octadecadienoic acid were not detected in larvae developing from photoperiods 10:14 and 14:10 (L:D). Larvae reared on 14:10 and 12:12 (L:D) had the highest percentage of 9-octadecenoic acid. In general, the percentages of fatty acids varied notably in the different larval categories. The results also confirm that the percent of fatty acids detected in the diapausing larvae varies significantly (F = 54.29; df = 6,35; P < 0.001), while the photoperiod regimes did not (F = 0.66; df = 5,36; P = 0.27). Nevertheless, there was a significant (F = 11.44; df = 11,30; P < 0.001) variation in the interaction of fatty acid and photoperiod regimes.

**Table 1:** Percent of fatty acids in non-diapausing (ND) and diapausing *P. interpunctella* larvae that had been exposed to different photoperiod regimes at 17 °C.

Fatty acids	Photoperiod regimes (L—light:D—dark)					
	ND	8L:16D	10L:14D	12L:12D	14L:10D	16L:8D
Methyl tetradecanoate	0.469	–	–	0.546	–	1.096
9-Hexadecenoic acid	1.79	3.533	1.14	2.918	7.821	7.514
Hexadecanoic acid	36.121	34.787	43.46	23.419	18.147	32.276
9,12-Octadecadienoic (Z,Z)-	21.389	23.792	18.15	28.41	27.408	23.1
9-Octadecenoic acid	33.842	32.098	35.32	42.36	36.919	29.303
Octadecadienoic acid	6.388	5.79	–	2.347	–	6.711
Methyl stearate	–	–	1.94	–	9.705	–

– Not available.

**Figure 7:**
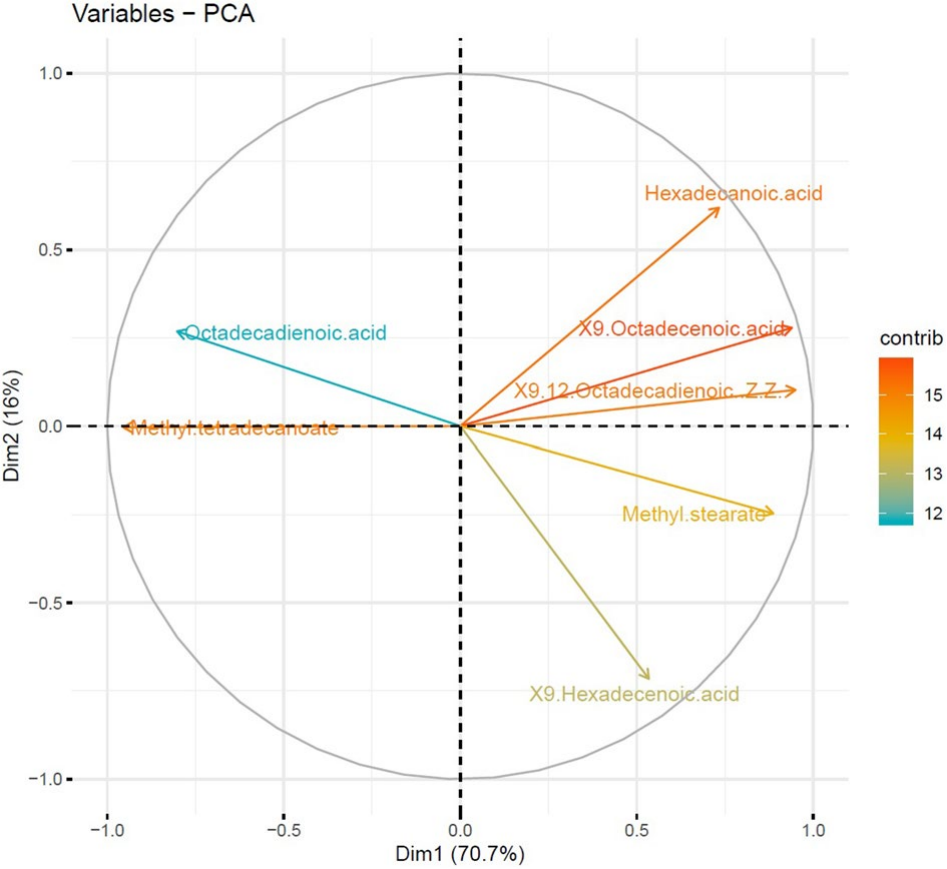
The PCA projection of individual metabolites on the correlation circle in photoperiodic induced diapause metabolite composition for fatty acids in *P. interpunctella* larvae.

**Figure 8:**
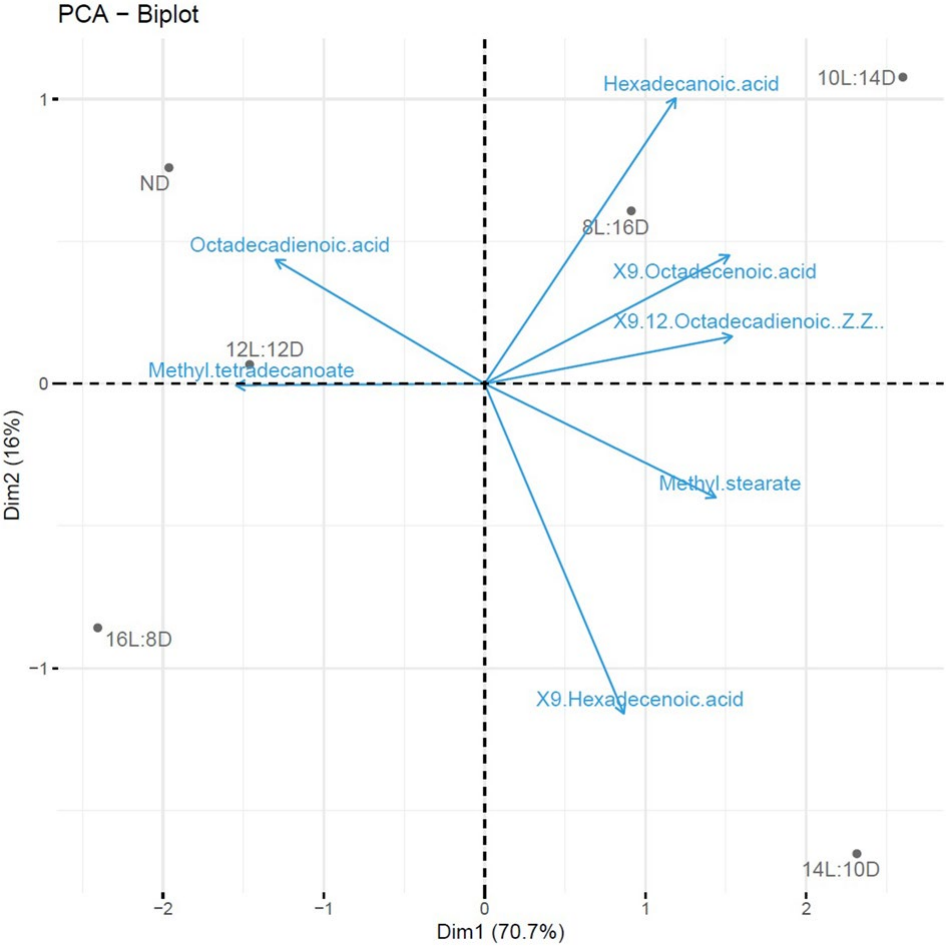
The PCA biplot projection of individual metabolites on the correlation circle in photoperiodic induced diapause metabolite composition for fatty acids in *P. interpunctella* larvae.

Fatty acid composition and ratios in diapausing larvae are summarized in Table 1 and Fig. 7. Approx. three quarters of the total variance was accounted for by PCA of the seven fatty acids. First principal component (PC) has large positive associations with 9-hexadecenoic and 9,12-octadecadienoic (Z,Z). The PCA biplot shows both PC scores of samples (dots) and loadings of variables (vectors) (Fig. 8). The PCA analyses indicated that the fatty acids octadecadienoic and methyl tetradecanoate exhibited positive correlation while they were negatively correlated with all other fatty acids. Moreover, most of the fatty acids including hexadecanoic acid, 9,12-octadecadienoic (Z,Z)-,9-octadecenoic acid, 9-hexadecenoic acid and methyl stearate showed positive correlation among them except 9-octadecenoic acid and methyl stearate. The biplot indicates that the photoperiods 8:16 and 10:14 (L:D) have more or less similar response patterns over variables compared to other photoperiods (Fig. 8).

## Discussion

Previous research indicated that a combination of short photophases with low temperatures can cause a rapid diapause induction of *P. interpunctella* larvae^16,17^. In general, photophases that were 13 h or shorter cause a quick diapause induction^19^, but diapause may not occur if the prevailing temperatures are high^19,57^. However, it has not been clarified whether the reduced activity of this species during the cold period of the year is related to increased diapause or decreased mobility due to reduced metabolism, although these two are likely related^11,58^. For *P. interpunctella* larvae, diapause is prevented at an increase of photoperiod to 16:8 (L:D), suggesting that temperature is less important than photoperiod in determining diapause^18^. Our results partially stand in accordance with the above observations, as the highest percentage of diapause induction was recorded at 12:12 (L:D). However, no significant differences were noted among the photoperiod levels tested here, indicating that the photophases evaluated were perhaps too narrow to detect differences that may have been observed if more photophases had been tested. Wang et al.^59^ systematically investigated the diapause induction and termination of small brown planthopper, *Laodelphax striatellus* (Fallén) by changing photoperiod and temperature. They also concluded that the temperatures ranging from 18 to 28 °C greatly influenced the incidence of diapause in *L. striatellus*. For instance, the photoperiodic response curve at 20 °C showed a gradual decline in diapause incidence in ultra-long nights, and continuous darkness resulted in 100% development. The different photoperiod regimes that were used in our experiments, in conjunction with exposure to 17 °C, might have affected diapause induction differently, as compared with the results reported by Bell^18^, but the diapause attributes of the different populations may account for the differences noted from other studies. Interestingly, we have shown little response to photoperiod with our *P. interpunctella* population*.* Still, we have noted different patterns of adult eclosion and sex ratio as a result of photoperiod, suggesting that different photoperiodic regimes may affect progeny production capacity of the exposed individuals.

Larval weight was notably increased at certain photoperiods. At the same time, at these photoperiods, the fatty acids composition was different, as compared with 8:16 and 14:10 (L:D). Differences in fatty acids among individuals that had been exposed to various photoperiods were previously noted for other species as well. For instance, for the silk moth, *Bombyx mori* (L.), Shimizu^60^ found that phosphatidylcholine of diapausing eggs contained more linolenic acid and less myristic acid than that of non-diapausing eggs. Similar reports have illustrated considerable differences between diapausing and non-diapausing individuals of the pink bollworm, *Pectinophora gossypiella* (Saunders), a feature linked to the timing of diapause termination^61,62^. The actual effect of the fatty acid composition on *P. interpunctella* larvae in diapause induction and termination is poorly understood, and requires additional investigation.

In a study of the tobacco moth, *Ephestia elutella* (Hübner), Bell^63^ underlined the importance of continuous rearing conditions in the laboratory in relation to diapause induction, which may not occur in “real world” conditions, in terms of regulation of biological cycle and succession of the generations. In this context, it has been reported that the diapause has been a major roadblock to developing control programs for many pests^64^. For instance, the sterile insect technique (SIT) and augmentative natural enemy control have been neither practical nor possible due to diapause responses that prevent or interfere with continuous mass rearing^64^. There are many examples in this regard, including the European cherry fruit fly^65^, the Apple maggot fly^66^, the Chinese citrus fruitfly^67^, the Russian melon fly^68^, and processionary moths^69^. Bloemi et al.^64^ noted that diapause induction, originally developed for individually reared codling moth, can be applied to the mass-rearing system used by the Sterile Insect Release (SIR) eradication program. The current research also suggests that there are some approaches that can potentially disrupt diapause and facilitate mass rearing. In this context, generalizations regarding the “optimum” conditions that are related with diapause should be avoided, as any “diapause”- related control strategy may not be accurate. On the other hand, considering that *P. interpunctella* is an ideal species to rear insect parasitoids, standardization of life table characteristics of a given population may be valuable to produce large numbers of parasitoids whenever these are needed^70,71^. Hence, considering our results, we have found that larvae of *P. interpunctella* can be used with success at various conditions, as the effect of different photoperiod regimes does not drastically affect diapause induction, when these larvae are reared at 17 °C. As Bell^18^ showed that diapause induction at a photophase of ≤ 13 h was not different between 20 and 25 °C, our study shows that pre-exposure at 17 °C might have widened the photoperiodic requirements towards this direction. Generally, the diapause of this species is classified as a long-day type^23,24^, while experiments with different photoperiodic regimes did not show a significant effect on diapause, unless the photoperiod was combined with specific thermo-period regimes^23,24,72^. It is well established that diapause in *P. interpunctella *is mostly related to scotophase rather than photophase, as constant scotophases provided similar diapause induction even when photophases are disrupted^73^. In this context, the critical scotophase varies from 8 to 12 h even when the photophase is kept at a 24-h duration^72^. This partially explains our results, considering that the scotophase used here was within these limitations.

## Conclusions

In summary, the results of the present study show specific photoperiodic associations in *P. interpunctella* larvae that seem to perform similarly across a wide range of photoperiods, provided that these individuals are exposed to 17 °C. Cold hardiness may be directly related to diapause induction^73,74^. As this temperature does not cause high larval mortality in this species^11,12,73^, it should be considered further for parasitoid rearings in mass production protocols.

## Methods

### Insect rearing

The colony of *P. interpunctella* used in the current study was collected originally from the local Rajshahi Municipal Food Market in 2014, and since then was cultured at the Post harvest Laboratory, Department of Zoology, Rajshahi University, Bangladesh. Moths were reared in 1 l glass jars on mixed standardized diet of corn meal, chick laying mash, chick starter mash, and glycerol at a volumetric ratio of 4:2:2:1, respectively^75^. The culture was maintained in an environmental chamber at 27 °C and 70% relative humidity (r.h.), with a photoperiod of 16:8 (L:D).

### Diapause induction of *P. interpunctella* larvae

**Figure 9:**
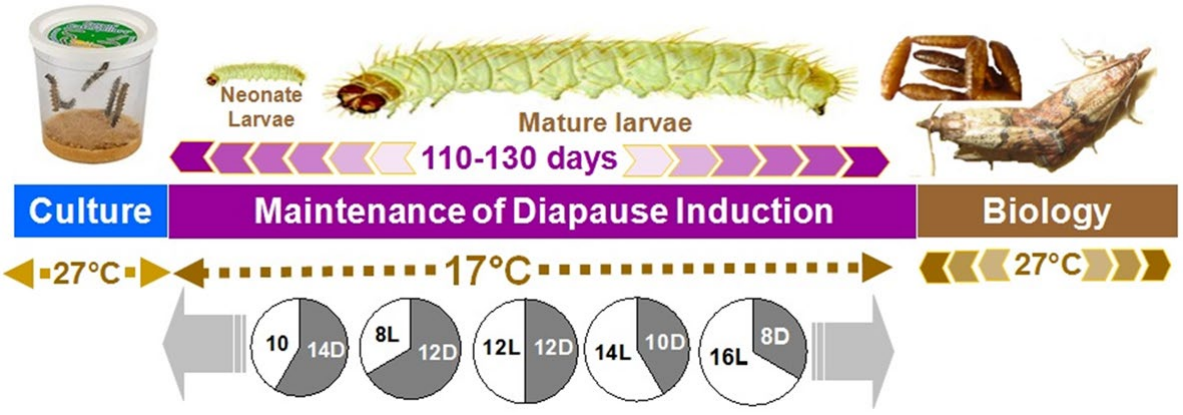
Schematic diagram for photoperiodic diapause induction in *P. interpunctella*.

Neonate larvae (1d old) of *P. interpunctella* were transferred individually into transparent plastic rearing trays (9.6″L × 4.1″W × 2.0″H) (HL-B025, Jiangsu, China) containing fifty small holes (2 ml) filled with food medium (5 g) at 17 °C and 60% r.h. Trays were covered with a transparent plastic sheet with tiny holes to allow exchange of air. Five photoperiods, i.e. 8:16, 10:14, 12:12, 14:10 and 16:18 (L:D) were tested for larval diapause induction. The experimental set up for photoperiodic diapause induction is presented in the schematic diagram of Fig. 9. The development of larvae was observed daily during the photophase. If a larva did not pupate after being reared for 40 d at 17 °C, it was considered to be in diapause. Diapausing larvae were also identified based on their extended larval developmental period, large size and yellowish color due to accumulated fat^19,21^. The number of larvae, pupae and moths per tray were recorded separately for each photoperiod regime and the percentage of diapausing larvae was then calculated. Mature larvae reared under each photoperiod regime were weighed using an electronic balance (FA-N/A-N, Shijiazhuang, China). Longevity, fecundity and eclosion of adults developing from diapausing and non-diapausing larvae were also recorded. The critical photoperiod is defined as the photoperiod that induces 50% of the maximum incidence of diapause^1^. There were three replications containing 50 larvae for each photoperiod regime. Under these conditions (17 °C and 60% r.h.), non-diapausing larvae pupated in the food and emerged as adults by day 60 after oviposition. As a light source, two 10-W daylight fluorescent bulbs were used and the photoperiodic cycles were controlled by a 24-h time switch^18^. The scotophase was controlled manually by wrapping cardboard boxes containing experimental material in a black polythene sheet.

### Protein analysis

Proteins in non-diapausing and diapausing mature larvae of *P. interpunctella *were measured according to Kjeldal method^76^. Percentages of nitrogen were transformed into protein content by multiplying a conversion factor of 5.3 as suggested^77,78^. There were three replicates, each with ten larvae for each of the photoperiod regimes.

### Fatty acid extraction and analysis

Lipids were extracted according to the method suggested by Folch et al*.*^79^. Twenty-five non-diapausing and diapausing mature larvae of *P. interpunctella* were homogenized in glass tubes and extracted three times with chloroform:methanol 2:1 (v/v). Supernatants were pooled and mixed with aqueous 0.88% KCl. After final centrifugation, the lower phase was collected and dried under a stream of N_2_. The lipid samples were processed for fatty acid analyses following methods described by Metcalf et al.^80^. The fatty acids of isolated lipids were methylated into reaction vials by refluxing with sodium methoxide (2%) for 10 min at 100 °C and then were transmethylated by refluxing with 2.175 ml boron trifluoride methanol 14% for 3 min at 100 °C. The fatty acid methyl esters (FAMEs) were extracted from the reaction vials three times with hexane, and concentrated. The fatty acids (FA) in the total lipids were determined as methyl ester derivatives by gas chromatography employing standard protocols^81^. The FAMEs were analyzed on a GCMS-QP2020 (SHIMADZU Co., Kyoto, Japan). The split ratio was 50:1 and 1 μl of solution was injected into the column. Helium was used as the carrier gas with flow rate of 1 ml/min. The oven temperature was kept at 140 °C for 5 min, increased at a rate of 3 °C/min to 240 °C, and held at 240 °C for 10 min. The injector and detector temperatures were maintained at 260 °C. The fatty acids were identified by comparing their retention times with those of the FAME standards under the same conditions. The fatty acid analyses were performed at Bangladesh Council of Scientific and Industrial Research (BCSIR) Laboratories, Rajshahi, Bangladesh.

### Statistical analysis

Assumptions of normality and homogeneity of variance were tested using Levene's^82^ method and indicated that the data should be arcsine transformed before the analysis. Then, the data were analyzed through ANOVA using the PROC GLMMIX (SAS Version 9.2, 2008)^83^, separately, for each of the scenarios indicated above. Means were compared by Tukey–Kramer HSD test at 5%. Untransformed means and standard errors are reported to simplify interpretation. Multivariate statistical procedures including principal component analysis (PCA) was employed to assess the differentiation as well as the relationship between fatty acids and photoperiod regimes. The PCA biplot was also designed to take multidimensional data sets and reduce their dimensions by determining one or more linear combinations of the variables. Moreover, PCA biplot showed both PC scores (PCs) of photoperiod regime (dots) and loadings of percent fatty acids (vectors). The PCs were sufficient to describe the essence of our data since the scree plot showed an ideal curve.

## References

[1] Tauber MJ, Tauber CA, Masaki S (1986). Seasonal Adaptations of Insects.

[2] Nylin S, Gotthard K (1998). Plasticity in life history traits. Annu. Rev. Entomol..

[3] Denlinger DL (2002). Regulation of diapause. Annu. Rev. Entomol..

[4] Aschoff J (1960). Exogenous and endogenous components in circadian rhythms. Cold Spring Harb. Symp. Quant. Biol..

[5] Bunning C (1960). Physiological Mechanism and Biological Importance of the Endogenous Diurnal Periodicity in Plants and Animals. Symposium Photoperiodism and Related Phenomena in Plants and ANIMALS.

[6] Pittendrigh CS (1960). Circadian rhythms and the circadian organization of living systems. Cold Spring Harbor Symp. Quant. Biol..

[7] Beck SD (1980). Insect Photoperiodism.

[8] Saunders DS (2002). Insect Clocks.

[9] Koštál V (2006). Eco-physiological phases of insect diapause. J. Insect Physiol..

[10] Bell CH (1994). A review of diapause in stored-product insects. J. Stored Prod. Res..

[11] Mohandass S, Arthur FH, Zhu KY, Throne JH (2007). Biology and management of *Plodia interpunctella* (Lepidoptera: Pyralidae) in stored products. J. Stored Prod. Res..

[12] Athanassiou CG, Arthur FH, Athanassiou CG, Arthur FH (2018). Bacterial insecticides and inert materials. Recent Advances in Stored Product Protection.

[13] Williams CM, Adkisson PL (1964). Physiology of insect diapause XIV. An endocrine mechanism for the photoperiodic control of pupal diapause in the oak silkworm *Antheraea**pernyi*. Biol. Bull..

[14] Wijayaratne LKW, Fields PG (2012). Effects of rearing conditions, geographical origin, and selection on larval diapause in the Indian meal moth, *Plodia interpunctella*. J. Insect Sci..

[15] Littlefair JE, Nunn KA, Knell RJ (2016). The development of a synthetic diet for investigating the effects of macronutrients on the development of *Plodia interpunctella*. Entomol. Exp. Appl..

[16] Johnson JA, Wofford PL, Gill RF (1995). Developmental thresholds and degree-day accumulations of Indian meal moth (Lepidoptera: Pyralidae) on dried fruits and nuts. J. Econ. Entomol..

[17] Bell CH, Walker DJ (1973). Diapause induction in *Ephestia elutella* (Hübner) and *Plodia interpunctella* (Hübner) (Lepidoptera, Pyralidae) with a dawn-dusk lighting system. J. Stored Prod. Res..

[18] Bell CH (1976). Factors governing the induction of diapause in *Ephestia elutella* and *Plodia interpunctella* (Lepidoptera). Physiol. Entomol..

[19] Mbata GN (1987). Studies on the induction of larval diapause in a Nigerian strain of *Plodia interpunctella* (Hübner) (Lepidoptera: Pyralidae). Insect Sci. Appl..

[20] Athanassiou CG, Buchelos CTh (2001). Detection of stored-wheat beetle species and estimation of population density using unbaited probe traps and grain trier samples. Entomol. Exp. Appl..

[21] Bell CH (1977). Toxicity of phosphine to the diapausing stages of *Ephestia elutella, Plodia interpunctella* and other lepidoptera. J. Stored Prod. Res..

[22] Bell CH (1982). Factors influencing the duration and termination of diapause in the Indian-meal moth, *Plodia**interpunctella*. Physiol. Entomol..

[23] Takeda, M. & Masaki, S. Photoperiodic control of larval development in *Plodia interpunctella*. In: *Proc. Joint U.S. Jap. Sem. Stor. Prod. Insect*, 186–201 (1976).

[24] Masaki S, Kikukawa S, Follett BK, Follett DE (1981). The diapause clock in a moth: response to temperature signals. Biological Clocks in Seasonal Reproductive Cycles.

[25] Kimura MT (1982). Effect of photoperiod on reproductive diapauses in *Drosophila testacea*. Experientia.

[26] Zaslavski VA, Fomenko RB (1983). Quantitative photoperiod perception in the aphid *Megoura**viciae* Buckt. (Homoptera: Aphididae). Entomol. Rev..

[27] Numata H (1985). Photoperiodic control of adult diapauses in the bean bug, *Riportus clavatus*. Mem. Fac. Sci. Kyoto Univ. Ser. Biol..

[28] Hori K (1987). Effects of stationary and changing photoperiods of nymphal growth of *Palomena angulosa* Motschulsky (Hemiptera: Pentatomidae). Appl. Entomol. Zool..

[29] Kimura MT (1990). Quantitative response to photoperiod during reproductive diapauses in the *Drosophila auraria* species-complex. J. Insect Physiol..

[30] Hardie J (1990). The photoperiodic counter, quantitative day-length effects and scotophase timing in the vetech aphid *Meguora viciae*. J. Insect Physiol..

[31] Spieth H, Sauer PS (1991). Quantitative measurement of photoperiods and its significance for diapause induction of *Pieris brassicae* (Lepidoptera, Pieridae). J. Insect Physiol..

[32] Denlinger DL (1986). Dormancy in tropical insects. Annu. Rev. Entomol..

[33] Hua A, Xue FS, Xiao HJ, Zhu XF (2005). Photoperiodic counter of diapause induction in *Pseudopidorus fasciata* (Lepidoptera: Zygaenidae). J. Insect Physiol..

[34] Danks HV (1987). Insect Dormancy: An Ecological Perspective.

[35] MacRae TH (2010). Gene expression, metabolic regulation and stress tolerance during diapause. Cell Mol. Life Sci..

[36] Williams KD, Schmidt PS, Sokolowski MB, Nelson RJ, Denlinger DL, Somers DE (2010). Photoperiodism in insect: molecular basis and consequences of diapause. Photo-periodism.

[37] Saunders DS, Nelson RJ, Denlinger DL, Somers DE (2010). Photoperiodism in insects: migration and diapause responses. Photo-periodism.

[38] Saunders DS (2010). Controversial aspects of photoperiodism in insects and mites. J. Insect Physiol..

[39] Goto SG, Shiga S, Numata H, Nelson RJ, Denlinger DL, Somers DE (2010). Photoperiodism in insects: percention of light and the role of clock genes. Photo-periodism.

[40] Koštál V (2011). Insect photoperiodic calendar and circadian clock: independence, cooperation or unity?. J. Insect Physiol..

[41] Denlinger DL, Lee RE (2010). Low Temperature Biology of Insects.

[42] Bale JH, Hayward SAL (2010). Insect overwintering in changing climate. J. Exp. Biol..

[43] Hahn DA, Denlinger DL (2007). Meeting the energetic demands of insects diapause: nutrient storage and utilization. J. Insect Physiol..

[44] Hahn DA, Denlinger DL (2011). Energetics of insect diapause. Annu. Rev. Entomol..

[45] Turunen S, Chippendale GM (1980). Proteins of the fat body of non-diapausing and diapausing larvae of the southwestern corn borer, *Diatraea grandiosella*: effect of juvenile hormone. J. Insect Physiol..

[46] Tan QQ (2017). Describing the diapause-preparatory proteome of the beetle *Colaphellus bowringi* and identifying candidates affecting lipid accumulation using isobaric tags for mass spectrometry-based proteome quantification (iTRAQ). Front. Physiol..

[47] Kikukawa S, Dillwith JW, Chippendale GM (1984). Characteristics of larvae of the south western corn borer, *Diatraea grandiosella,* obtained from populations present in tropical and temperature regions. J. Insect Physiol..

[48] Brown JJ, Chippendale GM (1978). Juvenile hormone and a protein associated with the larval diapause of the southwestern corn borer *Diatraea grandiosella*. Insect Biochem..

[49] Turunen S, Chippendale GM (1979). Possible function of juvenile hormone-dependent protein in larval insect diapause. Nature.

[50] Dillwith JW, Chippendale GM (1984). Purification and properties of a protein that accumulates in the fat body of prediapausing larvae of the southwestern corn borer, *Diatraea**grandiosella*. Insect Biochem..

[51] Roberts DB, Brock HW (1981). The major serum proteins of diptera larvae. Experientia.

[52] Telfer WH, Keim PS, Law JH (1983). Arylphorin, a new protein from *Hyalophora ceuropia*: comparison with calliphorin and manducin. Insect Biochem..

[53] Turunen S, Chippendale GM (1980). Fat body protein associated with the larval diapause of the southwestern corn borer, *Diatraea grandiosella:* synthesis and characteristics. Comp. Biochem. Physiol..

[54] Ashburner M, Bonner JJ (1979). The induction of gene activity in *Drosophila* by heat shock. Cell.

[55] Tzanakakis ME (1959). An ecological study of the Indianmeal moth *Plodia interpunctella* (Hübner) with emphasis on diapause. Hilgardia.

[56] Howe RW (1965). A summary of estimates of optimal and minimal conditions for population increase of some stored products insects. J. Stored Prod. Res..

[57] Prevett PF (1971). Some laboratory observation on the development of two African strains *Plodia interpunctella* (Hübner) (Lepidoptera: Phycitidae), with particular reference to diapause. J. Stored Prod. Res..

[58] Mason L, Hui YH (2003). Insects and mites. Food Plant Sanitation.

[59] Wang LF, Lin KJ, Chen C, Fu S, Xue FS (2014). Diapause induction and termination in the small brown planthopper, *Laodelphax striatellus* (Hemiptera: Delphacidae). PLoS ONE.

[60] Shimizu I (1992). Comparison of fatty acid compositions in lipids of diapause and non-diapause eggs of *Bombyx mori* (Lepidoptera: Bombycidae). Comp. Biochem. Physiol..

[61] Fife LC (1949). Studies of diapause of the pink bollworm in Puerto Rico. USDA Tech. Bull..

[62] Khalifa A, Elshaarawy MF, Shehata SM (2009). Incidence of diapause in pink bollworm, *Pectinophora**gossypiella* (Saund.). J. Appl. Entomol..

[63] Bell CH (1979). Factors influencing the duration and termination of diapause in the warehouse moth, *Ephestia elutella*. Physiol. Entomol..

[64] Bloemi S, Bloem KA, Fieldin LS (1997). Mass-rearing and storing codling moth larvae in diapause: a novel approach to increase production for sterile insect release. Entomol. Soc. B. C..

[65] Papanastasiou SA, Nestel D, Diamantidis AD, Nakas CT, Papadopoulos NT (2011). Physiological and biological patterns of a highland and a coastal population of the European cherry fruit fly during diapause. J. Insect Physiol..

[66] Meyers PJ (2016). Divergence of the diapause transcriptome in apple maggot flies: winter regulation and post-winter transcriptional repression. J. Exp. Biol..

[67] Chen Z (2016). Pupal diapause termination in *Bactrocera minax*: an insight on 20-hydroxyecdysone induced phenotypic and genotypic expressions. Sci. Rep..

[68] Saparmamedova NK (2004). A contribution to the knowledge of the melon fly *Myiopardalis**pardalina* Big. (Diptera, Tephritidae) in Turkmenia. Entomol. Rev..

[69] Salman MHR (2019). Termination of pupal diapause in the pine processionary moth *Thaumetopoea pityocampa*. Physiol. Entomol..

[70] Hasan MM, Yeasmin L, Athanassiou CG, Bari MA, Islam MS (2019). Using gamma irradiated *Galleria**mellonella* L. and *Plodia**interpunctella* (Hübner) larvae to optimize mass rearing of parasitoid *Habrobracon hebetor* (Say) (Hymenoptera: Braconidae). Insects.

[71] Hasan MM (2020). Mating attributes relating to parasitization and productivity in *Habrobracon hebetor* (Hymenoptera: Braconidae) rearing on host Indian meal moth (Lepidoptera: Pyralidae). J. Econ. Entomol..

[72] Kikukawa S, Masaki S (1984). Interacting effects of photophase and scotophase on the diapause response of Indian meal moth, *Plodia**interpunctella*. J. Insect Physiol..

[73] Fields P, Timlick B, Carvalho OM, Fields PG, Adler CS, Arthur FH, Athanassiou CG, Campbell JF (2010). The effect of diapause, cold acclimation and ice-nucleating bacteria on the cold-hardiness of *Plodia interpunctella*. Proceedings of the 10th International Working Conference on Stored Product Protection, Estoril, Portugal, 27 June–2 July 2010.

[74] Andreadis S, Athanassiou CG (2017). A review of insect cold hardiness and its potential in stored product insect control. Crop Prot..

[75] Phillips TW, Strand MR (1994). Larval secretions and food odors affect orientation in female *Plodia interpunctella*. Entomol. Exp. Appl..

[76] Jonas-Levi A, Martinez JI (2017). The high level of protein content reported in insects for food and feed is overestimated. J. Food Comp. Anal..

[77] Korel F, Balaban MO (2006). Microbial and sensory assessment of milk with an electronic nose. J. Food Sci..

[78] McCarthy MA, Meredith FI (1988). Nutrient data on chestnuts consumed in the United States. Econ. Bot..

[79] Folch JM, Lees M, Sloane-Stanley GH (1957). A simple method for the isolation and purification of total lipides from animal tissue. J. Biol. Chem..

[80] Metcalfe LD, Schmitz AA, Pelka JR (1966). Rapid preparation of fatty acid esters from lipids for gas chromatographic analysis. Anal. Chem..

[81] Käkelä R (2005). Fatty acid signatures in plasma of captive herring gulls as indicators of demersal or pelagic fish diet. Mar. Ecol. Prog. Ser..

[82] Levene H, Olkin I, Gleser LJ, Perlman MD, Press SJ, Sampson AR (1960). Contributions to Probability and Statistics. Essays in Honor of Harold Hetelling.

[83] SAS Institute (2008). SAS User’s Guide: Statistics.

